# Stabilization of OLFML1 via m^6^A Reader IGF2BP3 Drives CSC Characteristics Through Hedgehog Pathway Activation in CRC

**DOI:** 10.7150/ijbs.111032

**Published:** 2025-06-23

**Authors:** Yan Zhong, Jingfeng Liu, Wenwen Lin, Tian Peng, Lingfang Gao, Lan Shen, Ping Wang, Zhiyan Hu, Ting Long, Zuguo Li, Jingquan Liu

**Affiliations:** 1 Department of Pathology, Shenzhen Hospital, Southern Medical University, Shenzhen, Guangdong, China.; 2 Shenzhen Key Laboratory of Immunity and Inflammatory Diseases, Peking University Shenzhen Hospital, Shenzhen, China.; 3 Department of Pathology, School of Basic Medical Sciences, Southern Medical University, Guangzhou, Guangdong, China.

**Keywords:** colorectal cancer, m^6^A modification, OLFML1, IGF2BP3, cancer stemness, Hedgehog signaling pathway

## Abstract

Colorectal cancer (CRC) progression is closely associated with cancer stemness, which contributes to poor prognosis and therapeutic resistance. This study identifies OLFML1 as a key target accounting for CRC progression. High expression of OLFML1 promotes CRC cell proliferation and cancer stemness. As for mechanism study, we further revealed that IGF2BP3 as a critical up-stream regulator of OLFML1. Our study indicated that IGF2BP3 stabilizes OLFML1 mRNA through m^6^A modification, thereby enhancing its expression. In addition, IGF2BP3 prevents OLFML1 degradation via the ubiquitin-proteasome pathway. Clinically, this study demonstrated a positive association between IGF2BP3 and OLFML1 in CRC patient samples. High co-expression of IGF2BP3 and OLFML1 was significantly correlated with larger tumor size and advanced T stage. These findings highlight the IGF2BP3/OLFML1 axis as a potential driver of CRC stemness and Hedgehog pathway activation, offering promising prognostic and therapeutic targets for CRC management.

## Introduction

Colorectal cancer (CRC) is the third most commonly diagnosed malignancy worldwide and represents a significant global health burden, accounting for approximately 10% of the 19 million newly diagnosed cancer cases annually 1-3. Despite advances in early detection and therapeutic strategies, CRC remains a leading cause of cancer-related mortality. A major challenge in CRC management is the development of chemoresistance, which is a primary driver of tumor recurrence and poor clinical outcomes 4. The underlying molecular mechanisms contributing to chemoresistance, however, remain incompletely understood, necessitating further investigation to improve therapeutic efficacy and patient prognosis.

Emerging evidence suggests that cancer stemness, characterized by the presence of cancer stem cells (CSCs), plays a pivotal role in tumor heterogeneity, progression, and resistance to conventional chemotherapies 5-7. CSCs possess self-renewal and differentiation capabilities, enabling them to evade therapeutic interventions and contribute to tumor relapse 8-10. The interplay between cancer stemness and chemoresistance underscores the importance of elucidating the molecular pathways that regulate CSCs in CRC 11, 12. Identifying key regulators of CRC proliferation and stemness is therefore critical for the development of innovative and effective therapeutic strategies.

Olfactomedin-like 1 (OLFML1), a member of the olfactomedin (OLF) protein family, has recently garnered attention for its potential role in cancer biology 13-15. OLFML1 is characterized by an olfactomedin-like domain at its C-terminus and is predominantly expressed in tissues such as the small intestine, liver, heart, and spleen, while its expression is relatively low in normal colorectal tissues. Functionally, OLFML1 has been implicated in the formation of the extracellular matrix and osteoblast mineralization, suggesting its involvement in tissue homeostasis and structural integrity 16, 17. Notably, OLFML1 has been identified as a secreted glycoprotein that promotes cell cycle progression in human cancer cell lines under *in vitro* conditions. For instance, human OLFML1 (hOLFML1) has been shown to enhance the proliferation rate of HeLa cells 18. Despite these findings, the role of OLFML1 in cancer, particularly in CRC, remains poorly understood, and its potential contribution to tumor progression and stemness has yet to be fully elucidated.

Recent studies have highlighted the importance of the Hedgehog signaling pathway in regulating cancer stemness and tumor progression 19, 20. Aberrant activation of this pathway has been implicated in various malignancies, including CRC, where it contributes to CSC maintenance, proliferation, and chemoresistance 20, 21. However, the upstream regulators of Hedgehog signaling in CRC are not well-defined. In this context, understanding the molecular mechanisms by which OLFML1 interacts with the Hedgehog pathway could provide valuable insights into CRC pathogenesis and identify novel therapeutic targets.

In this study, we report for the first time that OLFML1 is significantly upregulated in CRC tissues and cell lines. Our findings reveal that OLFML1 expression is regulated by insulin-like growth factor 2 mRNA-binding protein 3 (IGF2BP3), a well-known RNA-binding protein implicated in cancer progression. Mechanistically, we demonstrate that IGF2BP3 stabilizes the expression of OLFML1 to activate the Hedgehog signaling pathway, thereby promoting CRC cell proliferation and enhancing cancer stemness. These results suggest that OLFML1 plays a critical role in CRC pathogenesis and may serve as a potential diagnostic biomarker and therapeutic target.

## Materials and Methods

### Clinical specimens

Clinical samples were collected from patients pathologically diagnosed with CRC at Southern Medical University Shenzhen Hospital. The study was conducted with the approval of the Ethics Committee of Southern Medical University Shenzhen Hospital as well.

### Cell lines and cell culture

The CRC cell lines SW620 (CCL-227), DLD1 (CCL-221), RKO (CRL-2577), HCT8 (CCL-244) and HEK293T cells (CRL-3216) were obtained from the American Type Culture Collection (ATCC). All CRC cells were cultured in RPMI 1640 medium (Gibco, USA) supplemented with 10% fetal bovine serum (FBS) (Gibco, USA) and maintained under standard conditions (5% CO_2_ and 95% atmosphere, 37°C). All the cell lines tested negative for mycoplasma.

### Immunohistochemistry (IHC)

Paraffin-embedded tumor tissues were sectioned and processed for immunohistochemical analysis as described before 3. The list of primary antibodies is included in Supplementary Table 4. The staining results were independently evaluated and scored by two pathologists. The percent positivity of antigen staining was scored as follows: 0 (0%), 1 (1-25%), 2 (26-50%), 3 (51-75%), and 4 (>75%). Staining intensity was graded on a 4-point scale: 0 (no staining), 1 (weak staining, light yellow), 2 (moderate staining, yellowish brown), and 3 (strong staining, brown). The antigen expression score was calculated by multiplying the percent positivity score by the staining intensity score, resulting in a range of 0 to 12. Antigen expression levels were categorized as follows: 0 (-), 1-4 (+), 6-8 (++), and 9-12 (+++). Tissues with scores of 0 (-) or 1-4 (+) were classified as the low-expression group, while those with scores of 6-8 (++) or 9-12 (+++) were classified as the high-expression group 3.

### Plasmids and lentiviruses

OLFML1^His^, IGF2BP3^Flag^ plasmids constructed with pcDNA3.1(+) cloning vector were purchased from Rui-biotech company (Guangzhou, China). CCoil, OLF-like, delSig truncations of His-OLFML1 and RRM+KH1-2, KH1-4, KH3-4 truncations of Flag-IGF2BP3 were constructed using PCR. Cells were transfected with 2 μg plasmid or the empty vector in Opti-MEM medium (Gibco) using Lipofectamine 3000 reagent (Invitrogen, Carlsbad, CA, USA) according to the manufacturer's instructions. A stable lentiviral vector with over-expression of OLFML1 (Gene Pharma, Suzhou, China) were constructed with human full-length cDNA. CRC cells were transduced with serial dilutions of lentiviral supernatant and selected by using 5 μg/mL puromycin for 2 weeks. The transfection efficiency was confirmed by RT-qPCR and western blots.

### siRNA transfection

siRNA targeting OLFML1 and IGF2BP3 siRNA were purchased from Gene Pharma (Suzhou, China). According to the manufacturer's protocol, siRNA duplexes were transfected into cells in an optiMEM medium using Lipofectamine 3000 reagent.

### RT-qPCR assay

Total RNA of CRC cells and CRC tissue were extracted using TRIzol reagent (Takara, 9109). RNA was reverse transcribed to cDNA with a Reverse Transcription Kit (Takara, D6110A). RT-qPCR analysis was performed using SYBR Green Master Mix (Takara, RR420). Primers used in this study were listed in Supplementary Table 5.

### Western blot

Total protein from cells or tissues was lysed by radioimmunoprecipitation assay (RIPA) buffer with phenylmethylsulphonyl fluoride (PMSF), protease inhibitors, and phosphatase inhibitors (FDbio, Hangzhou, Zhejiang, China). The protein was separated by sodium dodecyl-sulfate polyacrylamide gel electrophoresis (SDS-PAGE) and transferred onto polyvinylidene fluoride (PVDF) membranes. The PVDF membranes were blocked by 5% skim milk (FDbio) or 5% BSA (FDbio) for 1 h at room temperature and incubated with primary antibodies at 4 °C overnight. The membranes were then incubated with the corresponding secondary antibodies (FDbio) for 1 h at room temperature. Then protein was detected by ECL chemiluminescence solution (FDbio) and visualized by using the chemiluminescence detection system (Bio-Rad, California, USA). Antibodies used in this study were listed in Supplementary Table 4.

### Cell viability and colony formation assays

The proliferation of CRC cells in vitro was measured using Cell Counting Kit-8 (CCK-8, Dojindo). According to the manufacturer's instructions, cells were seeded into 96-well plates at 1 × 10^3^ per well in a final volume of 100 µL and cultured at 37 °C to obtain viable cells. For the colony formation assays, 800 cells per well were plated in six-well culture plates and cultured for 12-14 days at 37 °C in the presence of 5% CO2, then fixed with methanol and stained with crystal violet (0.5% solution, Beyotime). Three independent experiments were performed for both cell viability and colony formation tests. All the data was presented as means ± standard deviation.

### Immunofluorescence

Cells were seeded on confocal disks (Nest, Wuxi, China) and cultured for 24 h, washed with PBS, fixed with 4% formaldehyde for 30 min, permeabilized with 0.25% Triton X-100 for 8 min, blocked with goat serum (ZSGB-BIO) for 45 min, and then incubated with primary antibodies at 4 °C overnight. After being incubated with Alexa 488/594/355 conjugated secondary antibodies (ZSGB-BIO) or rhodamine phalloidin for 1 h and 4′,6-diamidino-2-phenylindole (DAPI) counterstaining for 10 min, the cells were observed and photographed by the Olympus confocal fluorescence microscope (FV1000).

### Tumor sphere formation assays

CRC cells were seeded into 6-well ultralow attachment plates at a density of 2 × 10³ cells per well and cultured in stem cell-conditioned medium. The medium consisted of 1640 medium (Invitrogen), 2% B-27 Supplement (Invitrogen), 20 ng/mL basic fibroblast growth factor (bFGF, PeproTech), 20 ng/mL epidermal growth factor (EGF, SinoBiological), 0.4% BSA (Sigma-Aldrich), and 5 µg/mL insulin (Sigma-Aldrich). The cells were incubated at 37 °C with 5% CO₂ and saturated humidity for 12-14 days. Culture suspensions were passaged every 7 days when spheroid diameters reached at least 50 μm. Images of the spheres were captured, and the number of cell spheres was counted. All experiments were performed at least three times, with three replicates for each treatment, and the results are presented as the mean ± standard deviation. To assess the self-renewal capacity of CRC cells, an in vitro limiting dilution assay (LDA) was conducted. Primary CSC spheres were collected, dissociated into single cells, and seeded into 96-well plates at densities of 5, 10, 20, 50, 100, or 200 cells per well. After 7 days, the formation of tumor spheres in each well was evaluated, and wells without tumor spheres were counted. Sphere formation efficiency was calculated using the Extreme Limiting Dilution Analysis (ELDA) tool (http://bioinf.wehi.edu.au/software/elda).

### Flow cytometry analysis

CRC cells were incubated with accutase and pipetted repeatedly to disperse into single cells. After washing twice with cold PBS, cells were centrifuged at 300×g for 5 min and re-suspended in binding buffer. CD133 antibody (12-1338-42, eBioscience, invitrogen) and Lgr5 antibody (50-9811-82, eBioscience, invitrogen) were added to the cell suspension and incubated for 30 min at 4 °C. Then, the cells were analyzed on a FACS flow cytometer according to the manufacturer's instructions. The results were analyzed by Flow-Jo software.

### EdU-DNA synthesis assay

An EdU Cell Proliferation Kit with Alexa Fluor 555 (MA0425, Meilun, Dalian) was used to detect proliferating cells after treatment with different concentrations of fatostatin for 24 h. The prewarmed EdU working solution was added to the treated cells for EdU labeling for 2 h. After removal of the medium, the cells were fixed for 30 min, and then, 500 μL of Click reaction solution was added to each well and incubated at room temperature for 30 min in the dark. Finally, 1 ml of Hoechst 33342 solution was added to each well, and the cells were incubated at room temperature for 10 min in the dark. After staining, a fluorescence microscope (Olympus BX51, Japan) was used to take the image.

### TUNEL assay

Adherent CRC cells were fixed in 4% paraformaldehyde for 0.5 h and treated in PBS containing 0.3% Triton X-100 for 10 min. The TUNEL mixture, including the TdT enzyme and fluorescence labeling solution was prepared according to the manual of the TUNEL assay kit (C1086, Beyotime). Then, 50 μL of the TUNEL mixture was mixed with the cell suspension and incubated at 37 °C for 1 h. Lastly, the cell nuclei were dyed with DAPI, and cells with TUNEL labeling were observed under the fluorescent microscope at 100×. Three random fields were selected from each group.

### Co-immunoprecipitation (Co-IP)

Total protein from the cells was lysed by RIPA buffer with PMSF, protease inhibitors, and phosphatase inhibitors (FDbio), followed by subjecting to IP with primary antibodies against OLFML1 or IGF2BP3 in a Protein A/G agarose IP Reagent (Bioworld, Minnesota, USA) at 4 °C overnight. The input was used as a positive control and normal rabbit immunoglobulin IgG was used as a negative control. The immunoprecipitated protein was detected by western blot.

### His pulldown assays

His pulldown assay was performed as described as before 3. Shortly, indicated bacteria-expressed OLFML1 his-tagged protein was purified and subsequently incubated with cell lysates from 293T cells transfected with IGF2BP3 overnight. The bound proteins were eluted and analyzed by western blot.

### Dual‑luciferase reporter gene assay

Experimental 293T cells were seeded into six-well plates and transfected with dual-luciferase reporter plasmids. After 48 h to 72 h of incubation, firefly and Renilla luciferase activities were quantified using the Dual-Luciferase Reporter Assay Kit (TransGene, Beijing, China), following the manufacturer's instructions. Firefly luciferase activity was normalized to Renilla luciferase activity to account for variations in transfection efficiency.

### Crosslinking immunoprecipitation

A crosslinking immunoprecipitation (CLIP) experiment was performed using a crosslinking immunoprecipitation and qPCR kit (BersinBio, Bes3014) following the manufacturer's instructions, to identify the specific OLFML1 mRNA sequence bound by IGF2BP3. After 16 h of treatment with 4-thiouridine, CRC cells were irradiated with 365 nm UV for 10 min to promote cross-linking. The cells were then lysed and enriched with IGF antibodies according to the kit instructions. The resulting precipitate was detected by RT-qPCR using primers for OLFML1.

### Methylated RNA immunoprecipitation

Total RNA was extracted using Trizol reagent (Thermo Fisher Scientific) and the concentration and integrity of total RNA were measured by Qubit RNA HS Assay and gel electrophoresis, respectively. The m^6^A RNA enrichment and RT-qPCR test were performed by E-GENE Tech Co., Ltd. Briefly, mRNA was isolated from 75 μg of total RNA using Dynabeads™ mRNA Purification Kit (Thermo Fisher Scientific). The purified mRNA was then fragmented in 1X RNA Fragmentation Buffer (Thermo Fisher Scientific) at 70 °C for 5 min. After purification, the fragmented RNA was used for enrichment with 5 μg of m^6^A antibody (Synaptic Systems) or IgG antibody which were previously incubated with 25 μL of protein A/G magnetic beads mixture (Thermo Fisher Scientific). The incubation was performed at 4 °C for 4 h with rotation. After immunoprecipitation, the magnetic beads were washed 3 times using IP buffer (150 mM NaCL, 10 mM Tris-HCL pH 7.5, 0.1% IGEPAL CA-630 in nuclease-free H_2_O) for 10 min of each time at 4 °C with rotation. Finally, the m^6^A modified mRNA fragments were purified with RNA Clean & Concentrator™ Kit (Zymo Research). After reverse transcription, cDNA from m^6^A-IP, IgG-IG and Input RNA were analyzed by real time PCR.

### Subcutaneous xenograft implantation models in nude mice

For tumorigenesis assays, CRC cells (2 × 10^6^ cells per mouse) were subcutaneously injected into the right dorsal flanks of nude mice (4-6 weeks of age, 18-20 g), which were obtained from the Animal Center of Southern Medical University. After 4 weeks, mice were sacrificed by cervical dislocation and the xenograft tumors were quickly harvested for histological study. The tumor volume was calculated according to the formula: Volume (mm^3^) = width^2^ (mm^2^) × length (mm)/2. Tumor volume was measured every 3 days.

## Results

### High levels of OLFML1 expression are associated with poor prognosis in CRC

**Figure 1:**
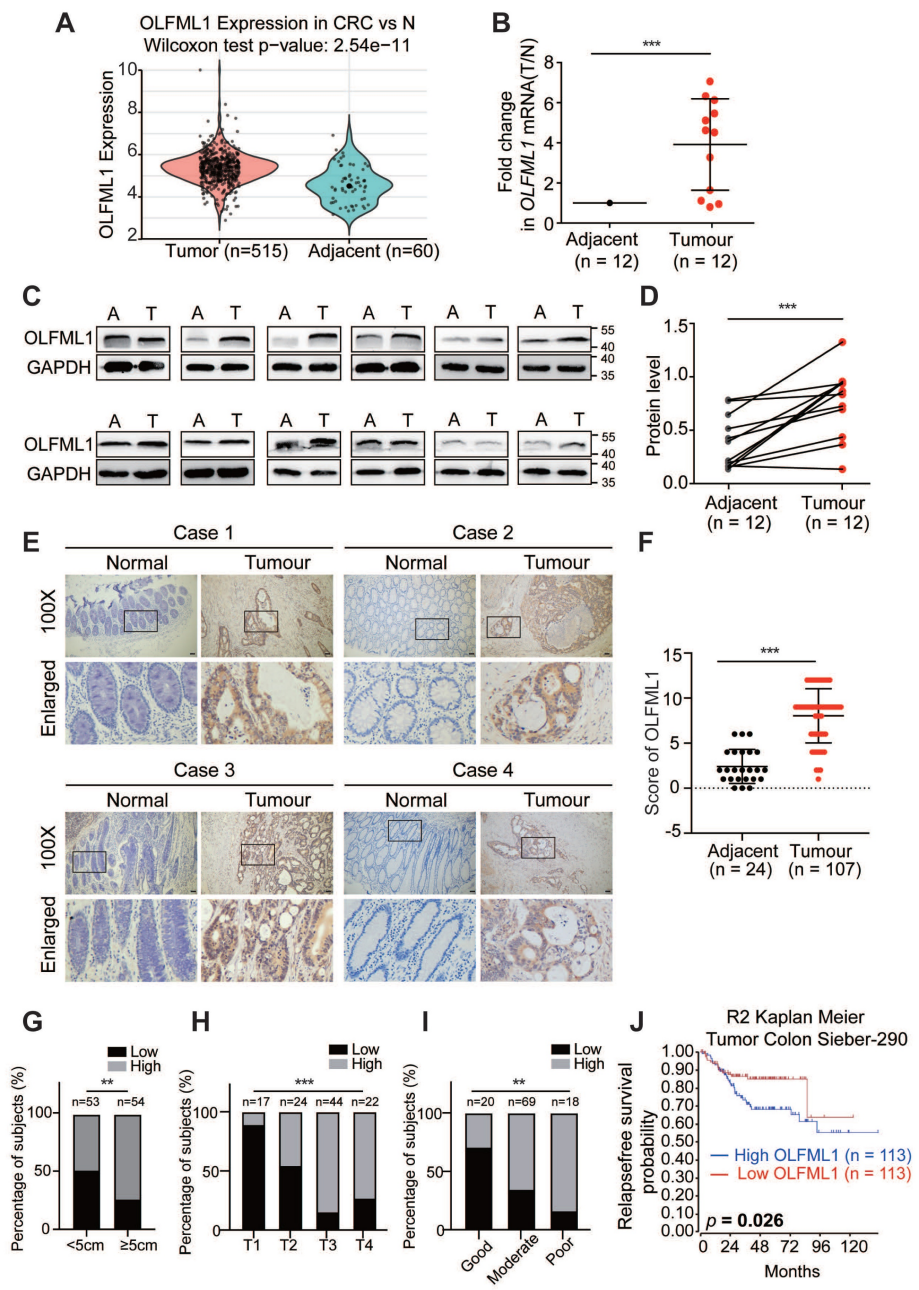
** OLFML1 is upregulated in CRC tissues and is positively correlated with poor prognosis in CRC patients. A** Analysis of OLFML1 expression in CRC tissues compared with adjacent normal tissues in the CRC microarray profiles (GSE71222, GSE21510, GSE17537, GSE128435, GSE64857 and GSE20970). **B** Fold change(T/N) of OLFML1 mRNA expression in 12 primary CRC tissues and adjacent normal tissues from the same patient, as determined by RT-PCR. **C, D** Immunoblot for OLFML1 protein expression in 12 human CRC tissues (T) and matched adjacent normal tissues (A) from the same patient. Quantification of protein levels were normalized to those of GAPDH were shown in the right panel. **E** Representative OLFML1 immunohistochemical staining images of adjacent normal tissue (Adjacent, n = 24) and tumor tissue (CRC, n = 107) samples (scale bar 20 μm). The magnified parts were displayed in the lower panel. **F** Immunohistochemical score of OLFML1 in adjacent tissues and CRC tumor tissues. **G-I** Percentage of high and low expression of OLFML1 in 107 CRC patients with different tumor size (G), T stages (H) and tumor differentiation (I). **J** Kaplan-Meier survival analysis of 226 CRC patients with low and high expression of OLFML1 from R2 Genomics Analysis and Visualization Platform (https://hgserver1.amc.nl/). **Data information**: Graphs report mean ± SD. Significance was assessed using 2-tailed Student's t-test, except for (G, H, I) where Chi-square test was used and (J) where log-rank test was used. ***P< 0.001, **P< 0.01, *P< 0.05.

To investigate the potential role of OLFML1 in the progression CRC, we integrated six colorectal cancer GEO databases (GSE71222, GSE21510, GSE17537, GSE128435, GSE64857 and GSE20970) to evaluate transcriptional levels of OLFML1. The results showed that OLFML1 expression was markedly higher in CRC tumor samples compared to normal mucosa (Fig. 1A and Fig. S1A). To validate these findings, we analyzed both mRNA and protein levels of OLFML1 in 12 pairs of primary CRC tissues and their corresponding adjacent normal tissues. The results confirmed significantly elevated OLFML1 expression in CRC tissues compared to adjacent normal mucosa (Fig. 1B, C, and D). Additionally, immunohistochemistry (IHC) was performed on paraffin-embedded CRC and normal tissue samples, further demonstrating higher OLFML1 protein expression in CRC tissues (Fig. 1E, F). This elevated expression of OLFML1 was positively correlated with larger tumor size, advanced T stage in the TNM classification, and poorer tumor differentiation (Fig. 1G-I and Supplementary Table S1). Kaplan-Meier survival analysis further revealed that high OLFML1 expression was significantly associated with worse prognosis in CRC patients (Fig. 1J and Fig. S1B). From above, our findings indicate that OLFML1 is upregulated in CRC tissues and is closely linked to tumor progression and unfavorable patient outcomes.

### OLFML1 promotes proliferation and inhibits apoptosis of CRC

**Figure 2:**
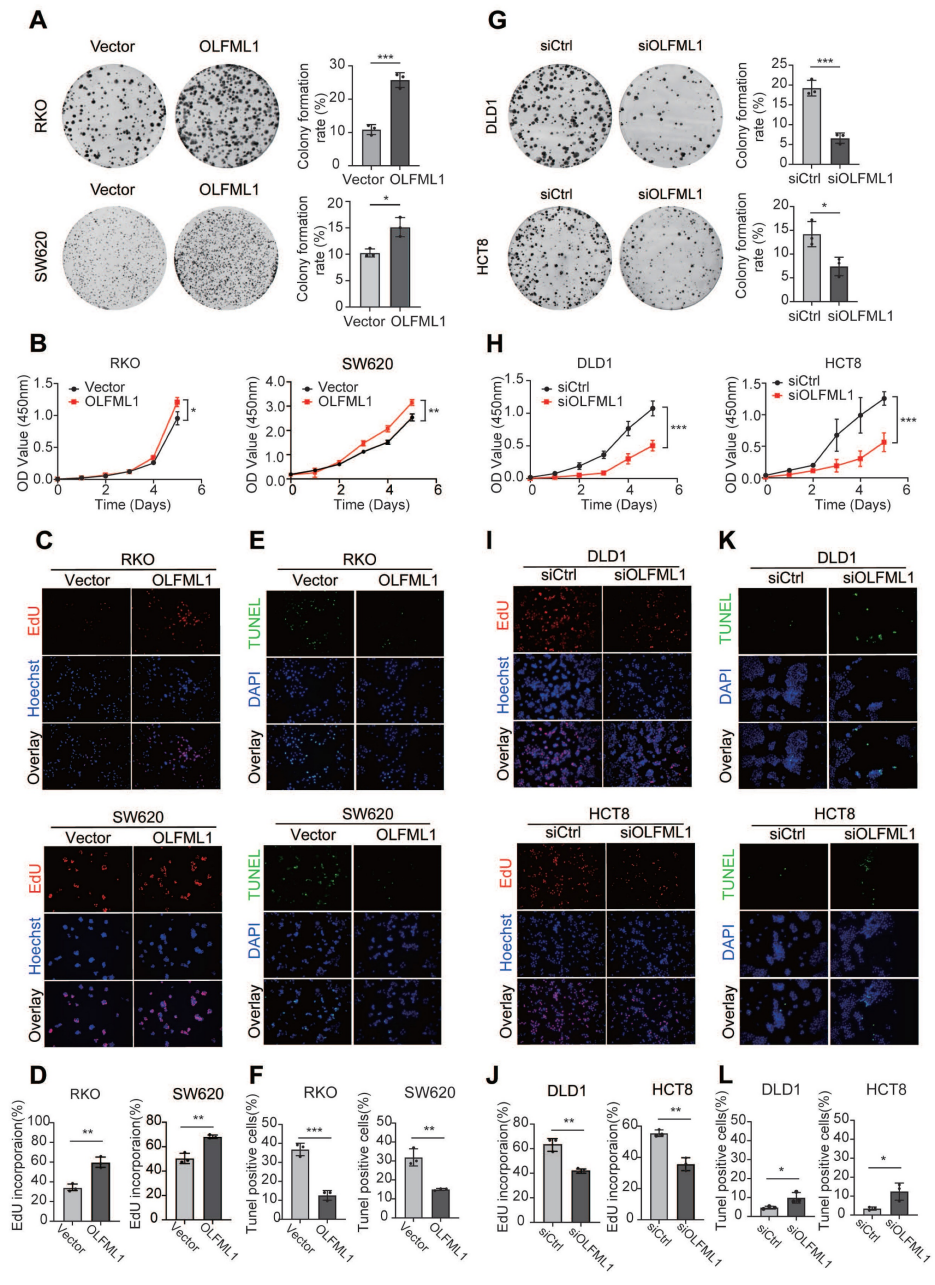
** OLFML1 promotes CRC cell proliferation. A** Colony formation assays were performed to determine the effects of OLFML1 on the growth of OLFML1-overexpressing CRC cells. The number of colonies (> 50 cells) was scored (n = 3). **B** CCK-8 assays were performed to determine the effects of OLFML1 upregulation on the proliferation of CRC cells (n = 4). **C, D** EdU assays were performed to determine the effects of OLFML1 upregulation on the proliferation of CRC cells (n = 3). **E, F** Tunel assays show the effects of OLFML1 upregulation on CRC cell apoptosis (n = 3).** G** Colony formation assays were performed to determine the effects of OLFML1 on the growth of OLFML1-depression CRC cells. The number of colonies (> 50 cells) was scored (n = 3). **H** CCK-8 assays were performed to determine the effects of OLFML1 depression on the proliferation of CRC cells (n = 4). **I, J** EdU assays were performed to determine the effects of OLFML1 depression on the proliferation of CRC cells (n = 3). **K, L** Tunel assays show the effects of OLFML1 depression on CRC cell apoptosis (n = 3). **Data information**: Graphs report mean ± SD, Significance was assessed using 2-tailed Student's t-test. ***P< 0.001, **P< 0.01, *P< 0.05.

**Figure 3:**
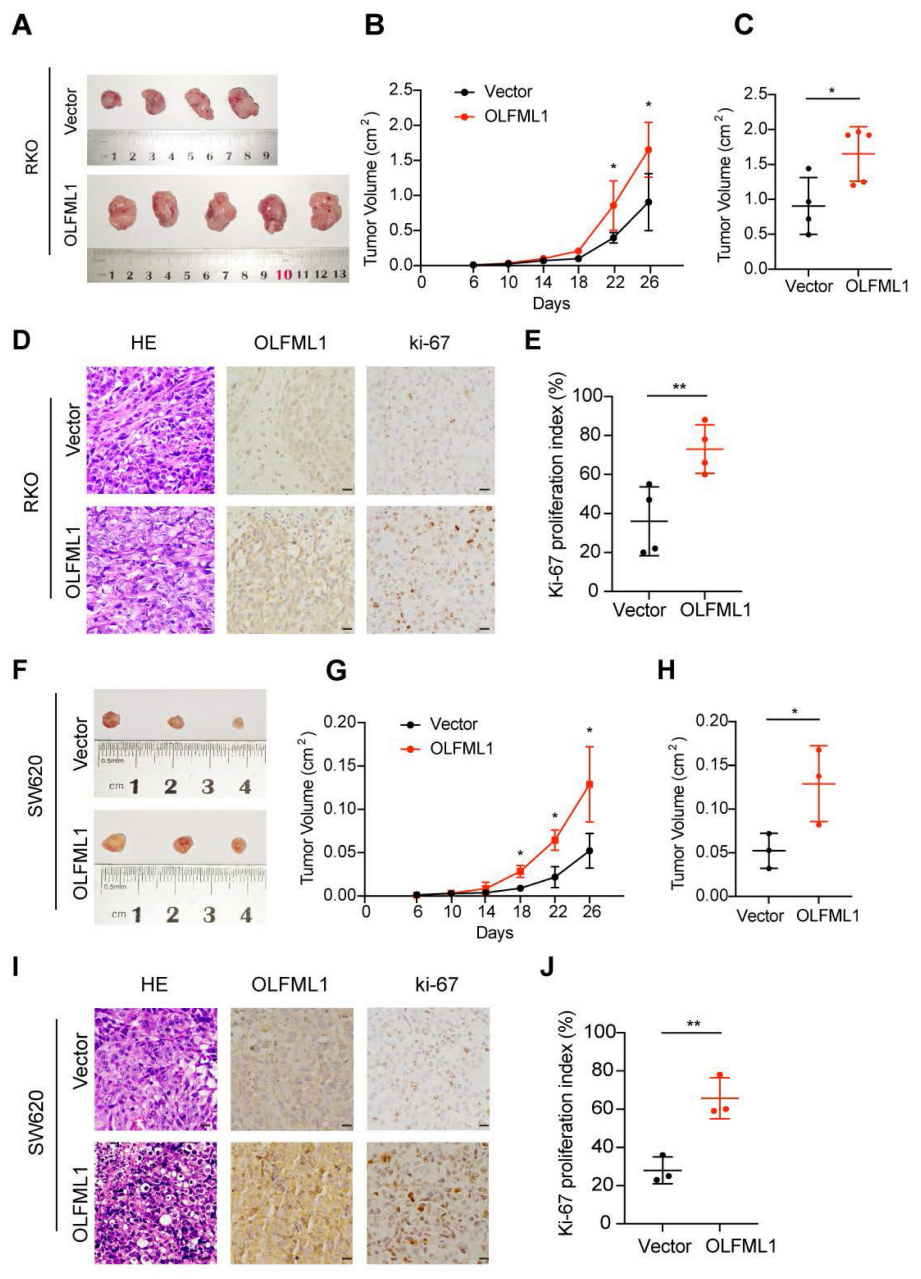
** OLFML1 promotes the proliferation of CRC in vivo. A** Vector and OLFML1-overexpressing RKO cells were injected subcutaneously into nude mice, as described in the Methods. **B** Tumor growth curves were obtained during twenty-six days. Tumors derived from RKO cells expressing OLFML1 grew significantly larger than tumors derived from control cells at the day point of 22^th^ and 26^th^. **C** Statistical analysis for tumor volume (cm^2^) of vector and OLFML1-overexpressing RKO cells at the sacrificed day. **D** Representative photographs of H&E, OLFML1 and Ki-67 immunohistochemistry staining of the primary tumor tissues from nude mice in RKO cells (scale bar 20 μm). **E** Statistical analysis for Ki-67 proliferation index between the group of vector and OLFML1-overexpressing RKO cells. **F** Vector and OLFML1-overexpressing SW620 cells were injected subcutaneously into nude mice, as described in the Methods. Twenty-six days later, tumors were removed and imaged. **G** Tumor growth curves were obtained during twenty-six days in the group of Vector and OLFML1-overexpressing SW620 cells. **H** Statistical analysis for tumor volume (cm^2^) of vector and OLFML1-overexpressing SW620 cells at the sacrificed day. **I** Representative photographs of H&E, OLFML1 and Ki-67 immunohistochemistry staining of the primary tumor tissues from nude mice in SW620 cells (scale bar 20 μm). **J** Statistical analysis for Ki-67 proliferation index between the group of vectors and OLFML1-overexpressing SW620 cells. **Data information**: Graphs report mean ± SD. Significance was assessed using 2-tailed Student's t-test. **P< 0.01, *P< 0.05.

To further investigate the function of OLFML1, we analyzed the mRNA and protein levels of OLFML1 in ten CRC cell lines (Fig. S1C-E) and successfully established OLFML1-overexpressing RKO and SW620 cell lines (Fig. S1F, G). Additionally, we silenced OLFML1 expression in DLD1 and HCT8 cells using three siRNA fragments. Among these, siOLFML1-3 was selected for subsequent experiments due to its superior knockdown efficiency compared to siOLFML1-1 and siOLFML1-2 (Fig. S1H, I). Functional assays revealed that OLFML1 overexpression significantly enhanced cell growth and colony formation in SW620 and RKO cells, as demonstrated by CCK-8 and colony formation assays (Fig. 2A, B). An EdU-DNA synthesis assay further confirmed that OLFML1 positively influences CRC cell proliferation (Fig. 2C, D). Conversely, TUNEL assays showed reduced apoptosis in OLFML1-overexpressing cells compared to control cells (Fig. 2E, F). In contrast, silencing OLFML1 in HCT8 and DLD1 cells significantly suppressed proliferation and increased apoptosis (Fig. 2G-L). We also examined the impact of OLFML1 on the efficacy of 5-fluorouracil (5-FU), a standard chemotherapy drug for CRC. Overexpression of OLFML1 enhanced the viability of 5-FU-treated SW620 cells, while OLFML1 knockdown reduced the viability of 5-FU-treated DLD1 cells (Fig. S1J, K). To validate these findings *in vivo*, we used subcutaneous xenograft models to assess the role of OLFML1 in tumor growth. Tumors derived from OLFML1-overexpressing cells were significantly larger than those from control cells at the time of dissection (Fig. 3A-C and 3F-H). IHC staining for Ki-67, a marker of cell proliferation, revealed a higher proliferation index in tumors formed by RKO/OLFML1 and SW620/OLFML1 cells compared to control tumors (Fig. 3D-E and 3I-J). In summary, these results demonstrate that OLFML1 is essential for CRC cell proliferation, anti-apoptotic activity, and tumor development, highlighting its potential as a key driver of CRC progression.

### OLFML1 promotes the stemness of CRC cells

**Figure 4:**
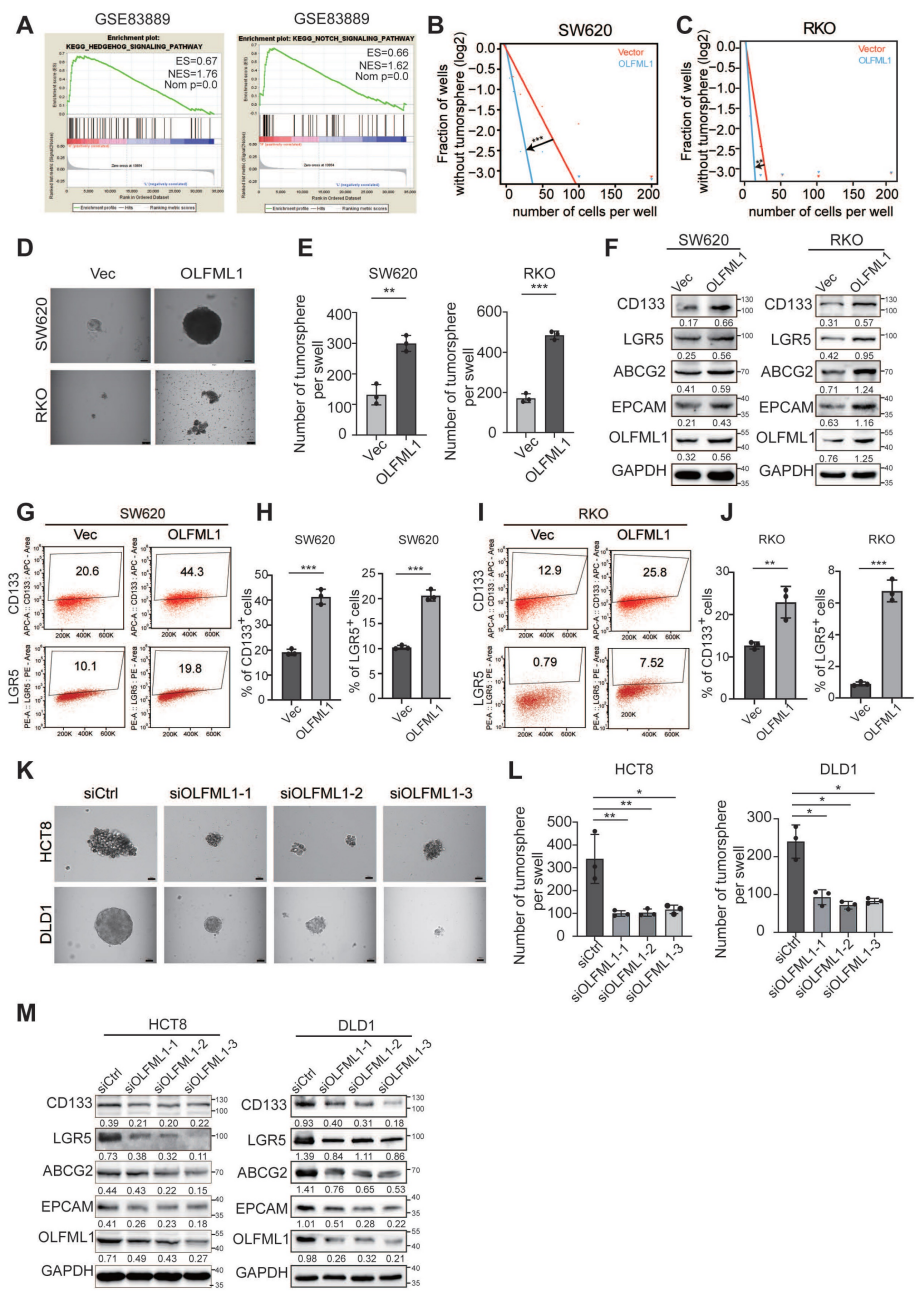
** Upregulation of OLFML1 enhances the stemness characteristics of CRC cells. A** The GSEA result indicates an enrichment of gene sets related to stemness-related signaling pathway in OLFML1 overexpression group of the CRC microarray profiles GSE83889. **B-C** The in vitro limiting dilution assay shows the effects of OLFML1 on the formation of CSC spheres (n = 12), likelihood ratio test. **D** Tumor sphere formation assays indicate that overexpressing of OLFML1 promotes the sphere formation of CSC cells. Scale bar represents 50 μm. **E** The number of tumorsphere in the control and OLFML1-overexpressing CRC cells (n = 3). **F** Western blot assays were performed the effects of OLFML1 on the expression of stem cell markers in CRC cells. **G-J** The number of CD133 + or LGR5 + cells was evaluated in the vector, and OLFML1 overexpression CRC cells by flow cytometry (n = 3). **K** Tumor sphere formation assays indicate that downregulation of OLFML1 decreased the sphere formation of CSC cells. Scale bar represents 50 μm. **L** The number of tumorsphere in the control and OLFML1-depressing CRC cells (n = 3). **M** Western blot assays were performed the effects of OLFML1 knockdown on the expression of stem cell markers in CRC cells. **N** Western blots assays demonstrate the effect of silencing OLFML1 on expression of Hedgehog pathway markers in CRC cells. **Data information**: Graphs report mean ± SD. Significance was assessed using 2-tailed Student's t-test. ***P< 0.001, **P< 0.01, *P< 0.05.

**Figure 5:**
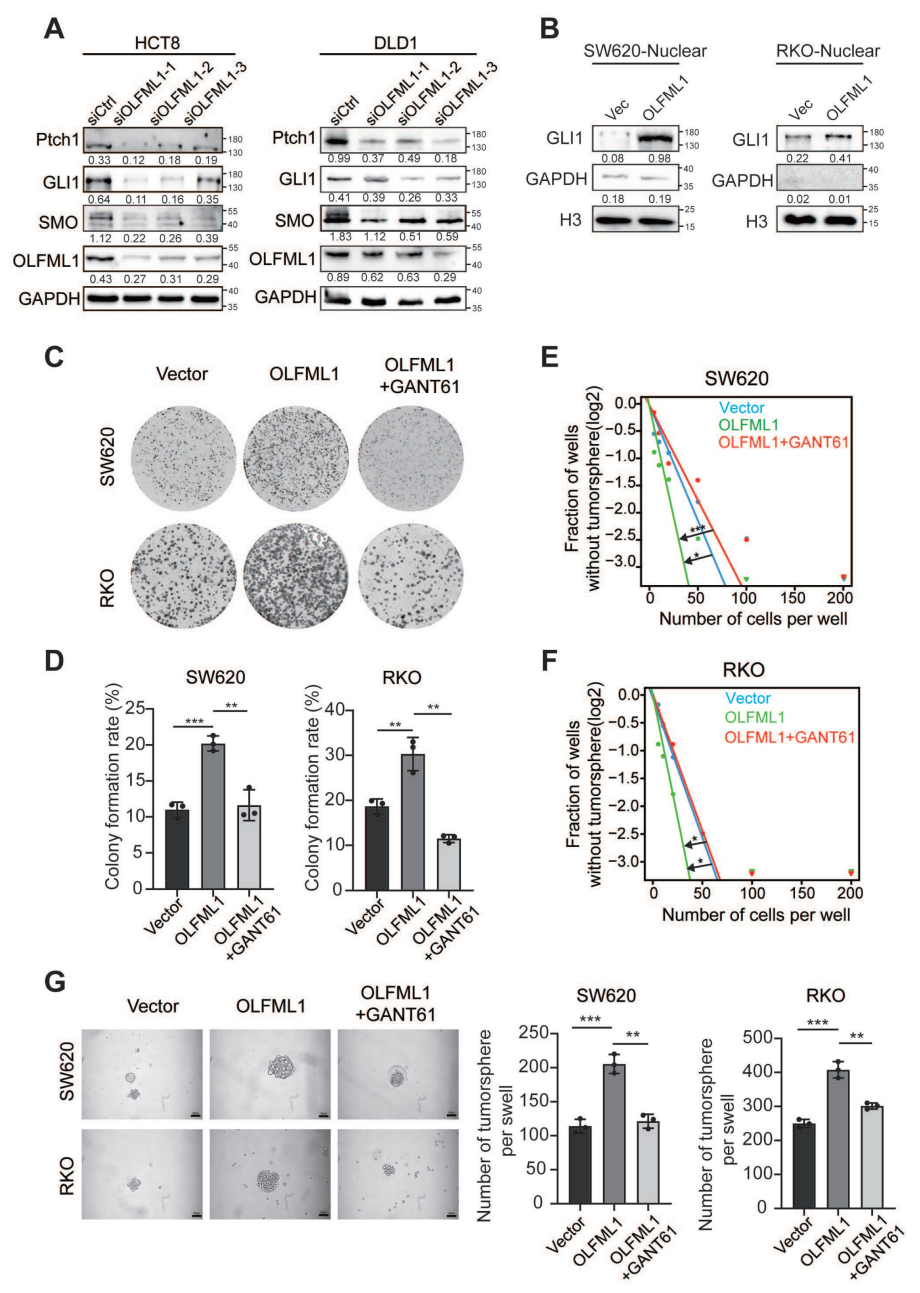
** OLFML1 promotes the proliferation and stemness of colorectal cancer cells by activating the hedgehog signaling pathway. A** Western blots assays demonstrate the effect of silencing OLFML1 on expression of Hedgehog pathway markers in CRC cells. **B** The GLI1 protein expression in nuclear of the indicated CRC cells was detected by western blotting. The quantification of nuclear GLI1 was normalized to H3. **C, D** Colony formation assays were performed in CRC cells treated with Vector, OLFML1 overexpression, and both OLFML1 overexpression and GLI1 inhibitors (GANT61). **E, F** The in vitro limiting dilution assay showed that OLFML1 increases the formation proportion of CSC spheres, simultaneously treat with GANT61 reverses this effect (n = 12), likelihood ratio test. **G** Tumor sphere formation assays indicate that OLFML1 enhances the formation of CSC spheres, simultaneously treat with GANT61 reverses this effect (n = 3). **Data information**: Graphs report mean ± SD. Significance was assessed using 2-tailed Student's t-test. ***P< 0.001, **P< 0.01, *P< 0.05.

To explore the mechanisms by which OLFML1 regulates proliferation in CRC, we performed gene set enrichment analysis (GSEA) on microarray data from GSE83889, GSE20842, GSE35297, GSE37178, GSE37182. The GSEA results revealed that stemness-related pathways, including the Hedgehog and NOTCH signaling pathways, were significantly enriched in OLFML1-overexpressing CRC tissues (Fig. 4A and Fig. S2A). To further investigate the role of OLFML1 in maintaining cancer stemness, we conducted *in vitro* limiting dilution assays. Overexpression of OLFML1 significantly enhanced the self-renewal capacity of SW620 and RKO cells (Fig. 4B, C). Additionally, OLFML1-overexpressing cells formed larger and more numerous CSC spheres compared to control cells (Fig. 4D, E). Western blot analysis confirmed that OLFML1 overexpression increased the expression of stemness markers, including CD133, LGR5, ABCG2, and EPCAM, in both RKO and SW620 cells (Fig. 4F). Flow cytometry further demonstrated an elevated number of CD133^+^ and LGR5^+^ cells in OLFML1-overexpressing SW620 and RKO cells (Fig. 4G-J). Conversely, silencing OLFML1 in HCT8 and DLD1 cells resulted in fewer and smaller CSC spheres and reduced the expression of stemness markers, as shown by western blot and RT-qPCR analysis (Fig. 4K-M and Fig. S2B, C). Consistent with the GSEA findings from the other four GSEA database (GSE20842, GSE35297, GSE37178, GSE37182), knockdown of OLFML1 also suppressed the protein expression of Ptch1, GLI1, and SMO, which are key components of the Hedgehog signaling pathway (Fig. 5A). Although we enriched the NOTCH signaling pathway, the enhanced expression of OLFML1 did not affect the changes of key proteins in NOTCH signaling pathway (Fig. S2D).

GLI1, a key protein in the Hedgehog signaling pathway, undergoes nuclear translocation, which is essential for pathway activation. Therefore, we extracted nuclear proteins and verified that OLFML1 overexpression led to increased nuclear GLI1 expression (Fig. 5B). This finding suggests that OLFML1 activates the Hedgehog signaling pathway by facilitating GLI1 nuclear translocation. Subsequently, we treated OLFML1-overexpressing cells with the Hedgehog pathway inhibitor GANT61, which in turn, OLFML1-induced cell colony formation and enhanced cancer stemness was suppressed (Fig. 5C-G). These results strongly suggest that OLFML1 plays a pivotal role in maintaining CRC stemness by regulating stemness-related pathways, particularly the Hedgehog signaling pathway. This highlights OLFML1 as a potential therapeutic target for disrupting cancer stemness in CRC.

### Interaction validation between OLFML1 and IGF2BP3

**Figure 6:**
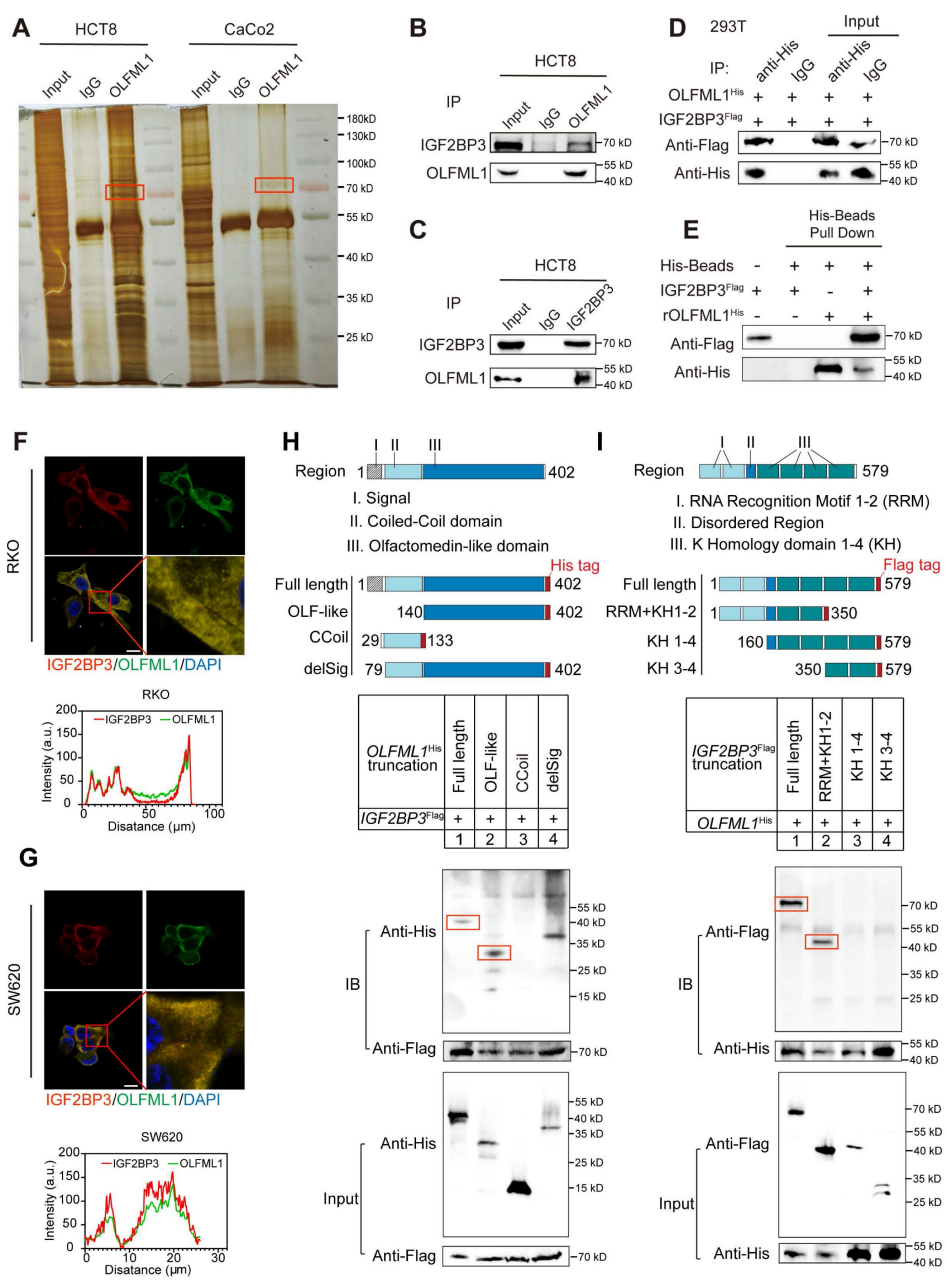
** OLFML1 interacts with IGF2BP3 directly. A** HCT8 and CaCo2 cells were lysed and subjected to immunoprecipitation with anti-OLFML1 antibody or rabbit IgG using silver staining. The red boxes represented for the detected bands. **B, C** Endogenous OLFML1 and IGF2BP3 were immunoprecipitated in HCT8 cells. **D** Exogenous OLFML1^His^ and IGF2BP3^Flag^ were immunoprecipitated in 293T cells. **E** Direct binding of rOLFML1^His^ and IGF2BP3^Flag^ using His pulldown assay. **F, G** OLFML1 colocalized with IGF2BP3 in RKO and SW620 cells. scale bar 50 μm. **H** Diagrammatic representation of OLFML1 and its truncated forms. Based on sequence and structure analyses, region I (signal peptide), region II (Coiled-Coil domain) and region III (Olfactomedin-like domain) were indicated. 293T cells were transfected with indicated constructs subjected to immunoprecipitation with anti-Flag (against IGF2BP3). Immunoblot analysis was performed with anti-Flag or anti-His (against OLFML1). The red boxes represented for the pull-down bands. **I** Diagrammatic representation of IGF2BP3 and its truncated forms. Based on sequence and structure analyses, region I (RNA Recognition Motif), region II (Disorder Region) and region III (K Homology domain) were indicated. 293T cells were transfected with indicated constructs subjected to immunoprecipitation with anti-His (against OLFML1). **Data information:** Graphs report mean ± SD.

To uncover the molecular mechanisms by which OLFML1 promotes CRC proliferation and stemness, an immunoprecipitation (IP) assay was performed. A differential protein band at approximately 70 kDa was identified and analyzed using liquid chromatography-mass spectrometry (LC-MS) (Fig. 6A). Among the proteins pulled down by OLFML1, IGF2BP3 had the highest score (Fig. S3A). The interaction between endogenously expressed OLFML1 and IGF2BP3 was confirmed in HCT8 and CaCo2 cells (Fig. 6B-C and S3B). Additionally, the interaction between exogenously expressed His-tagged OLFML1 (OLFML1^His^) and Flag-tagged IGF2BP3 (IGF2BP3^Flag^) was validated in HEK293T cells (Fig. 6D). To further determine whether OLFML1 directly binds to IGF2BP3, a His pull-down assay was performed using purified His-tagged OLFML1 protein and IDA-Nickel His beads. The assay confirmed that OLFML1 directly binds to IGF2BP3 but not to the His control (Fig. 6E). Immunofluorescence (IF) assays further demonstrated significant co-localization of OLFML1 and IGF2BP3 in the cytoplasm of CRC cells (Fig. 6F, G). These findings strongly suggest that OLFML1 interacts with IGF2BP3 directly in CRC cells.

OLFML1 is known as a secreted protein containing an OLF-like domain and a coiled-coil domain on its cytoplasmic facet. To further investigate the binding domain, three truncations of OLFML1 with His-tags were constructed and transfected into HEK293T cells along with Flag-tagged IGF2BP3. Co-immunoprecipitation (Co-IP) experiments revealed that IGF2BP3 specifically co-immunoprecipitated with the Olfactomedin-like domain of OLFML1 (amino acids 140-402) (Fig. 6H). To identify the domain of IGF2BP3 critical for OLFML1 binding, three IGF2BP3^Flag^ truncations were constructed. Co-IP assays demonstrated that OLFML1 co-immunoprecipitated with the RNA Recognition Motif domain (RRM domain, amino acids 1-156) of IGF2BP3 (Fig. 6I). In summary, these results demonstrate that OLFML1 directly interacts with IGF2BP3 in CRC cells, with the Olfactomedin-like domain of OLFML1 binding to the RRM domains of IGF2BP3. This interaction may play a critical role in mediating the effects of OLFML1 on CRC proliferation and stemness.

### Upregulation of IGF2BP3 promotes the stemness of CRC cells

**Figure 7:**
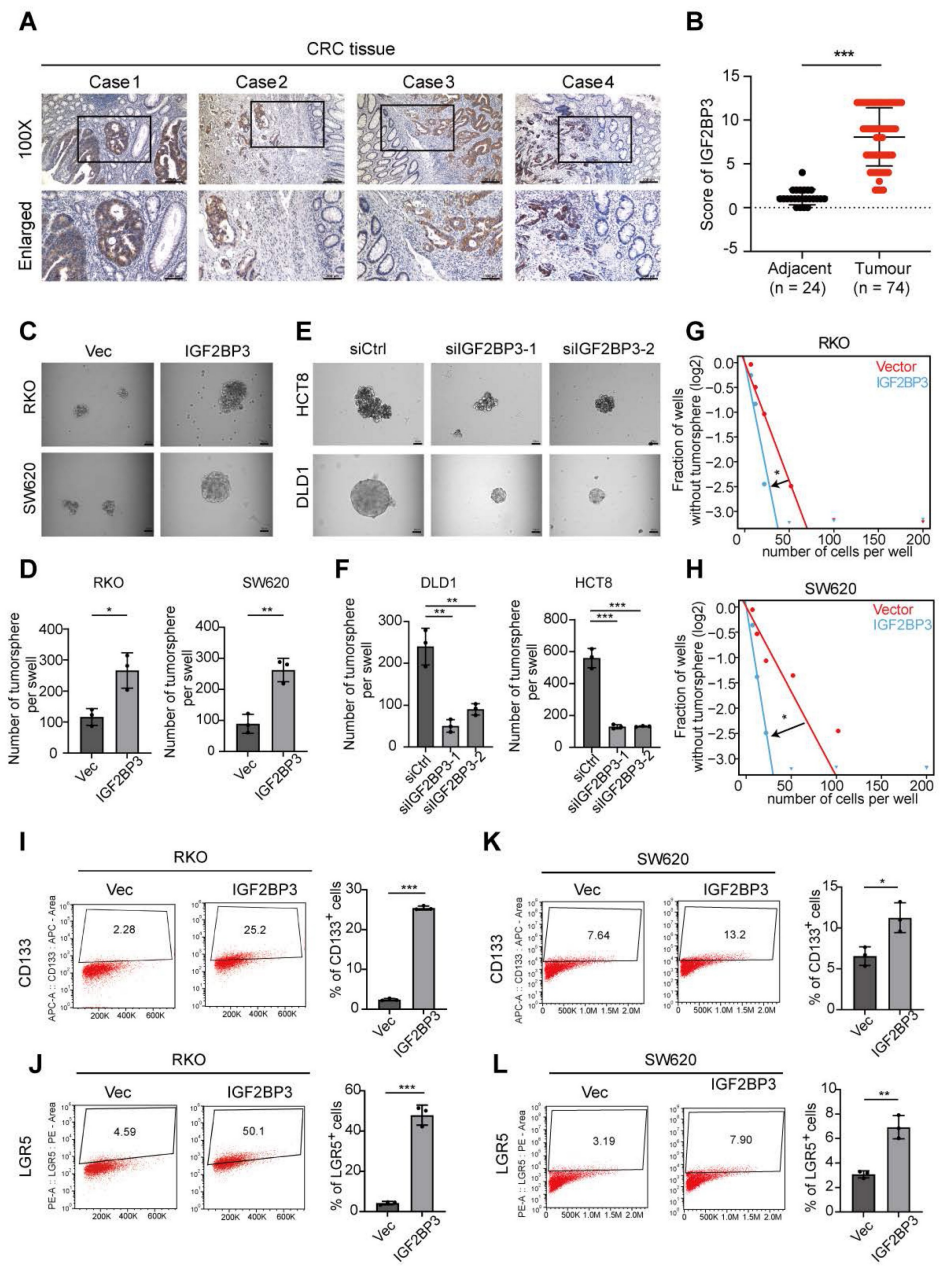
** IGF2BP3 is upregulated in CRC tissues and enhances the stemness characteristics of CRC cells. A** Representative IGF2BP3 immunohistochemical staining images of adjacent normal tissue (Adjacent, n = 24) and tumour tissue (CRC, n = 74) samples. The magnified parts were displayed in the lower panel. **B** Immunohistochemical score of IGF2BP3 in adjacent tissues and CRC tumour tissues. **C, D** Tumor sphere formation assays indicate that overexpression of IGF2BP3 in RKO and SW620 cells promotes the sphere formation of CSC cells. Scale bar represents 50 μm (n = 3). **E, F** Tumor sphere formation assays indicate that IGF2BP3 downregulation in DLD1 and HCT8 cells reduces CSC sphere formation. The Scale bar represents 50 μm (n = 3). **G, H** The in vitro limiting dilution assay shows the effects of IGF2BP3 on the formation of CSC spheres (n = 12), likelihood ratio test. **I-L** The number of CD133 + or LGR5 + cells was evaluated in the control and IGF2BP3 overexpressing RKO and SW620 CRC cells by flow cytometry (n = 3). **Data information:** Graphs report mean ± SD. Significance was assessed using 2-tailed Student's t-test. ***P< 0.001, **P< 0.01, *P< 0.05.

To confirm the upregulation of IGF2BP3 protein levels in CRC, its expression was analyzed in whole tissue sections from CRC samples and adjacent normal tissues using IHC (Fig. 7A). IHC imaging and scoring revealed that IGF2BP3 expression was significantly higher in 74 CRC tissues compared to 24 adjacent non-tumor tissues (Fig. 7A, B). This upregulation was positively correlated with larger tumor size, indicating that IGF2BP3 may contribute to CRC progression (Table S2). To investigate the functional role of IGF2BP3 in CRC proliferation and stemness, colony formation assays, CCK-8 assays and spheroid formation assays were performed. Overexpression of IGF2BP3 in RKO and SW620 cells significantly promoted proliferation ability and the formation of larger CSC spheroids (Fig. 7C, D and Fig. S4A-D). Conversely, knockdown of IGF2BP3 in HCT8 and DLD1 cells suppressed CSC spheroid formation (Fig. 7E, F). Additionally, overexpression of IGF2BP3 enhanced the self-renewal capacity of RKO and SW620 cells, as demonstrated by limiting dilution assays (Fig. 7G, H). To further assess the impact of IGF2BP3 on the stem cell population in CRC cells, flow cytometry was used to measure the expression of stemness markers LGR5 and CD133. IGF2BP3-overexpressing RKO and SW620 cells exhibited increased cell surface expression of LGR5 and CD133 compared to control vector-transfected cells (Fig. 7I-L). These findings highlight IGF2BP3 as a critical regulator of stemness in CRC, suggesting that it plays a pivotal role in promoting CSC properties and potentially contributing to CRC progression.

### IGF2BP3 acts as an upstream regulator of OLFML1 transcription

**Figure 8:**
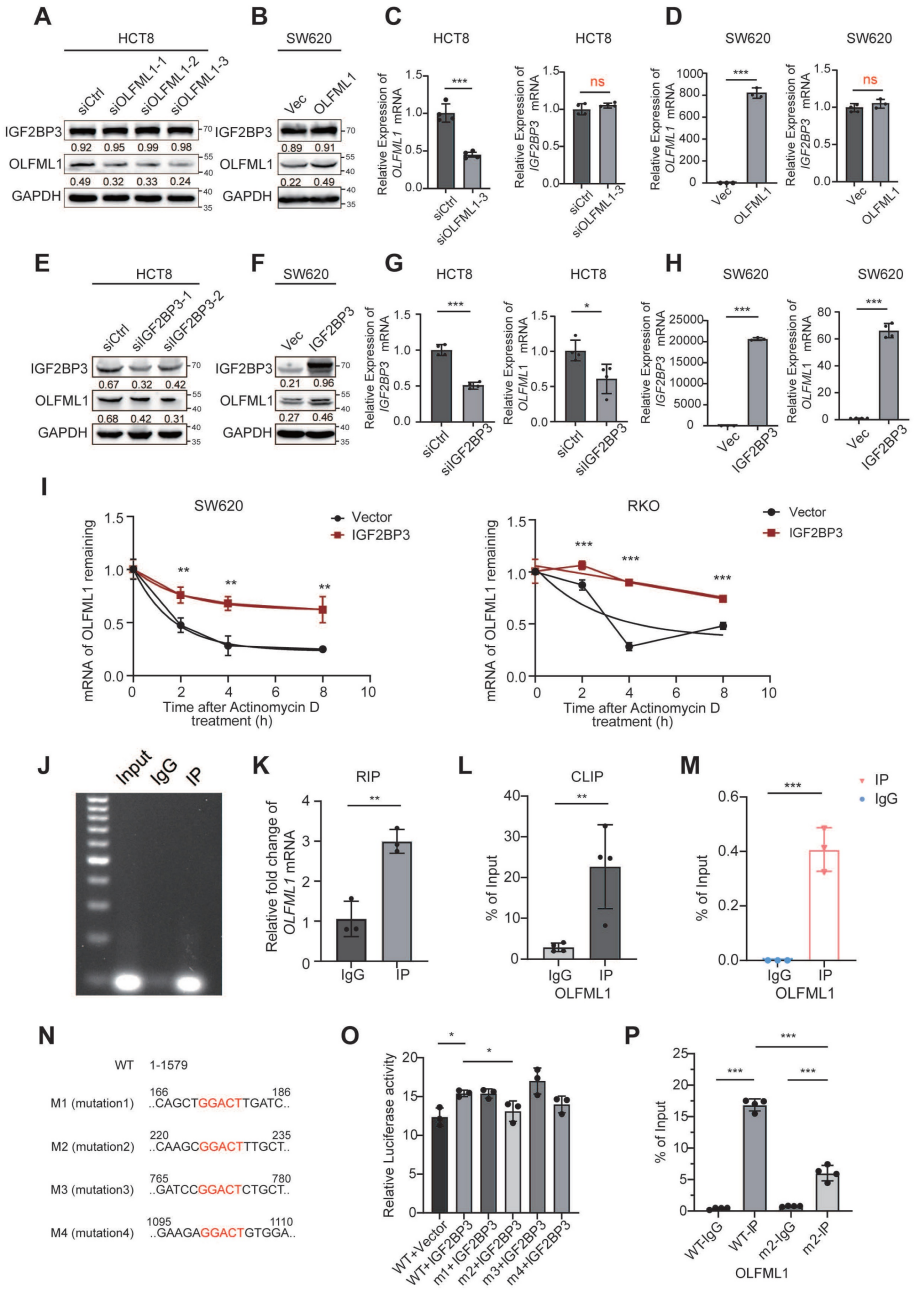
** IGF2BP3 enhances the mRNA and protein stability of OLFML1. A, B** Western blot shows IGF2BP3 protein expression levels in HCT8/siCtrl and HCT8/siOLFML1 cells (A) or SW620/Vector and SW620/OLFML1 cells (B). **C, D** The mRNA expression levels of IGF2BP3 in HCT8/siCtrl and HCT8/siOLFML1 cells (C) or in SW620/Vector and SW620/OLFML1 cells (D). **E, F** Western blot shows OLFML1 protein expression levels in HCT8/siCtrl and HCT8/siIGF2BP3 cells (E) or SW620/Vector and SW620/IGF2BP3 cells (F). **G, H** The mRNA expression levels of OLFML1 in HCT8/siCtrl and HCT8/siIGF2BP3 cells (G) or in SW620/Vector and SW620/IGF2BP3 cells (H). **I** The remaining OLFML1 mRNA in the control and IGF2BP3 upregulation conditions detected by Real-time q-PCR in SW620 and RKO cells. **J, K** Agarose gel electrophoresis and Real-time q-PCR was used to detect nucleic acids amplified by OLFML1 primers after RNA immunoprecipitation with anti-IGF2BP3 antibody or rabbit IgG. **L** CLIP assay was used to verify the binding between IGF2BP3 and OLFML1 mRNA. **M** The relative mRNA level of OLFML1 was detected by Real-time q-PCR after M^6^A methylation antibody pull-down. **N** Schematic representation of mutations in the OLFML1 sequence for the investigation of the roles of m6A in OLFML1 mRNA expression. **O** Overexpression of IGF2BP3 can promote the expression of luciferase in OLFML1 WT plasmid, but this promoting effect was abolished when the M2 site of OLFML1 was mutated, as verified by a dual-luciferase reporter assay in HEK293T cell. **P** Compared with the wild-type (WT) plasmid, the amount of m6A enriched by OLFML1 was reduced after mutation of the M2 site, as verified by a MeRIP assay in HEK293T cells. **Data information:** Graphs report mean ± SD. Significance was assessed using 2-tailed Student's t-test. ***P< 0.001, **P< 0.01, *P< 0.05.

Next, to explore the regulatory relationship between IGF2BP3 and OLFML1, western blot and RT-qPCR were conducted. The results showed that overexpression or knockdown of OLFML1 did not affect the expression of IGF2BP3 (Fig. 8A-D and Fig. S5A). However, IGF2BP3 positively regulated both the mRNA and protein levels of OLFML1 (Fig. 8E-H and Fig. S5B). Notably, by using CHX to inhibit intracellular protein degradation, we observed that overexpression of IGF2BP3 extended the degradation time of OLFML1, confirming that IGF2BP3 enhances the stability of OLFML1 at the protein level (Fig. S5C). Furthermore, we found that the reduction in OLFML1 expression caused by IGF2BP3 knockdown was significantly rescued by MG132 treatment (Fig. S5D-E). This suggests that IGF2BP3 inhibits OLFML1 degradation via the ubiquitin-proteasome pathway, thereby positively regulating OLFML1 protein expression.

Given that IGF2BP3 is an RNA-binding protein, its effect on OLFML1 mRNA stability was examined. SW620 and DLD1 cells were treated with actinomycin D to block mRNA synthesis, and it was observed that IGF2BP3 overexpression enhanced the stability of OLFML1 mRNA (Fig. 8I). The direct interaction between IGF2BP3 and OLFML1 mRNA was confirmed using RIP-qPCR and CLIP, with IgG serving as a negative control (Fig. 8J-L).

To investigate the mechanism by which IGF2BP3 binds to OLFML1 mRNA, the online tool SRAMP (http://www.cuilab.cn/sramp) was used to predict m^6^A modification sites in OLFML1 mRNA, identifying high-confidence m^6^A sites (Fig. S5F). MeRIP assays were then performed in CRC cells using m^6^A-specific antibodies. The results demonstrated that the methylation antibody significantly enriched OLFML1 mRNA compared to IgG (Fig. 8M), indicating that m^6^A modifications are present on OLFML1 mRNA. Based on the predicted OLFML1 m^6^A sites identified by the SRAMP prediction tool, we selected four sites for deletion mutations and constructed four OLFML1 plasmids with mutated m^6^A sites (Fig. 8N) for subsequent experiments. The dual-luciferase reporter assay revealed that OLFML1-WT plasmid binds directly to IGF2BP3. However, mutating the second methylation site (M2, bases 225-229) significantly weakened the binding between IGF2BP3 and OLFML1 (Fig. 8O). These results confirm that the interaction between IGF2BP3 and OLFML1 depends on the M2 m^6^A site of OLFML1. Validation through MeRIP demonstrated that mutating the M2 site dramatically reduced the amount of OLFML1 pulled down by the m^6^A antibody (Fig. 8P).

These findings suggest that IGF2BP3 regulates OLFML1 expression through two mechanisms: (1) by stabilizing OLFML1 mRNA via m^6^A modification and (2) by preventing OLFML1 protein degradation through inhibition of the ubiquitin-proteasome pathway. This dual regulatory role highlights IGF2BP3 as a critical upstream regulator of OLFML1 in CRC.

### IGF2BP3 regulates CRC stemness and progression through OLFML1

**Figure 9:**
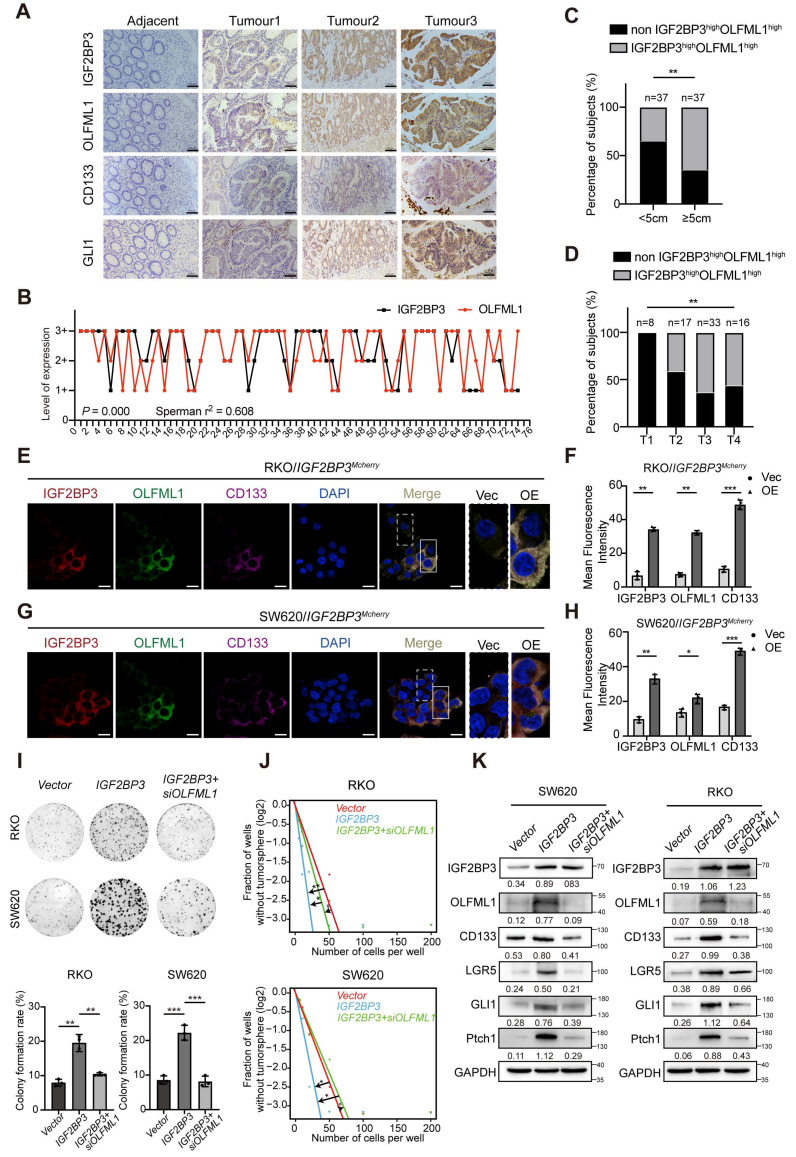
** IGF2BP3 is correlated with OLFML1 expression in CRC and enhances the stemness of CRC cells. A** Representative IGF2BP3, OLFML1, CD133 and GLI1 immunohistochemical staining images in serial sections from the same tumour tissue (CRC, n = 74) samples, scale bar 100 μm. **B** Spearman's correlation analyses between IGF2BP3 and OLFML1 expressions in CRC tissues. The level of 1+ represented for the score of 1-4, and 2+ represented for the score of 6-8, and 3+ represented for the score of 9-12. Spearman r = 0.608, n = 74. **C, D** Percentage of IGF2BP3^high^ OLFML1^high^ (simultaneously high expression of both IGF2BP3 and OLFML1) or non-IGF2BP3^high^ OLFML1^high^ (non-high co-expression of IGF2BP3 and OLFML1) in 74 patients with different tumor size (C) and T stages (D). **E-H** Immunofluorescence images of OLFML1 and CD133 in RKO/IGF2BP3^Mcherry^ (E) and SW620/IGF2BP3^Mcherry^ (G) cells. The magnified parts of vector cells (dotted line) and IGF2BP3^Mcherry^ overexpression cells (solid line) were respectively displayed. Mean fluorescence intensity levels of IGF2BP3, OLFML1 and CD133 from magnified parts in the left panel (n = 3 cells analysed per group). **I** Colony formation assays were performed in CRC cells treated with Vector, IGF2BP3 overexpression, and both IGF2BP3 overexpression and OLFML1 knockdown. **J** The in vitro limiting dilution assay showed that IGF2BP3 enhances the formation of CSC spheres, simultaneously knockdown of OLFML1 reverses this effect (n = 12), likelihood ratio test. **K** Western blot assays demonstrate that overexpression of IGF2BP3 promotes expression of stem cell markers in CRC cells and hedgehog pathway-related proteins, while simultaneously knockdown of OLFML1 reverses this effect. **Data information:** Graphs report mean ± SD. Significances were assessed using 2-tailed Student's t-test, except for (B) where Spearmen's correlation test was used and (C, D) where Chi-square test was used. ***P< 0.001, **P< 0.01, *P< 0.05.

Clinical analysis revealed a significant positive correlation between the expression levels of IGF2BP3 and OLFML1, OLFML1 and CD133, IGF2BP3 and CD133 as well as OLFML1 and GLI1, IGF2BP3 and GLI1 in serial sections from the same CRC patients (Fig. 9A-B and S6A-D). Furthermore, simultaneous high expression of IGF2BP3 and OLFML1 was strongly associated with larger tumor size and advanced T stage in the TNM classification (Fig. 9C-D and Table S3). Immunofluorescence (IF) staining confirmed that IGF2BP3-overexpressing RKO and SW620 cells exhibited high expression of OLFML1 and CD133 proteins, with significant overlap and co-localization (Fig. 9E-H). To investigate the role of OLFML1 in IGF2BP3-mediated regulation of stemness, RKO and SW620 cells overexpressing IGF2BP3 were treated with siRNA targeting OLFML1 (si-OLFML1), while RKO and SW620 cells with IGF2BP3 knockdown were treated with OLFML1 over-expression. Colony formation assays and tumor sphere formation assays revealed that OLFML1 knockdown significantly reduced the colony-forming ability and stemness promoted by IGF2BP3 overexpression. Moreover, OLFML1 over-expression successfully reversed the inhibition of proliferation and stemness caused by IGF2BP3 downregulation (Fig. 9I, J and Fig. S7A-F). Additionally, OLFML1 knockdown suppressed IGF2BP3-induced upregulation of stemness markers CD133 and LGR5, as well as Hedgehog signaling pathway components GLI1 and Ptch1 in both SW620 and RKO cells (Fig. 9K). In conclusion, these findings underscore the critical role of IGF2BP3 in regulating CRC stemness and progression through OLFML1. The simultaneous high expression of IGF2BP3 and OLFML1 is associated with poor prognosis in CRC patients, as it promotes stemness, tumor growth, and progression. This highlights the IGF2BP3-OLFML1 axis as a potential therapeutic target for CRC.

## Discussion

In this study, we identified OLFML1 as a novel regulator of CRC progression and stemness. Our findings demonstrate that OLFML1 is significantly upregulated in CRC tissues and cell lines, and its expression is regulated by IGF2BP3. Mechanistically, we revealed that OLFML1 activates the Hedgehog signaling pathway, a key regulator of cancer stemness 20, to promote CRC cell proliferation and enhance cancer stem cell properties. These results provide new insights into the molecular mechanisms underlying CRC progression and suggest that OLFML1 may serve as a potential diagnostic biomarker and therapeutic target. This study represents the first comprehensive investigation into the role of OLFML1 in CRC, highlighting its critical involvement in tumor biology and its potential clinical relevance.

Despite the significant findings, this study has certain limitations that warrant further exploration. One key area requiring additional investigation is the regulatory relationship between IGF2BP3 and OLFML1. IGF2BP3 is known to function as an m^6^A reader, binding to m^6^A-modified mRNA to regulate its stability, translation, or degradation 22-24. While our results suggest that IGF2BP3 stabilizes OLFML1 mRNA expression by binding directly to m^6^A modification sites, the precise mechanism remains unclear. Future studies employing m^6^A sequencing or other molecular biology assays are needed to confirm the role of m^6^A modification in this regulatory axis. Additionally, it will be important to determine whether other m^6^A-related proteins, such as methyltransferases or demethylases, are involved in modulating OLFML1 expression. Clarifying these mechanisms will provide a deeper understanding of how IGF2BP3 regulates OLFML1 and its downstream effects on CRC progression. Furthermore, other than mRNA stabilization, this study also indicated that IGF2BP3 enhances OLFML1 protein stability by preventing its ubiquitination-mediated degradation. This dual regulatory mechanism—stabilizing OLFML1 at both the mRNA and protein levels—underscores the critical role of IGF2BP3 in maintaining OLFML1 expression and function in CRC. However, the precise molecular interactions between IGF2BP3 and the ubiquitination machinery remain to be elucidated. Future research should focus on identifying the specific E3 ligases or de-ubiquitinates involved in this process and determining how IGF2BP3 interferes with these pathways. Addressing these questions will provide a more comprehensive understanding of the IGF2BP3-OLFML1 regulatory axis and its contribution to CRC progression.

The potential of OLFML1 as a therapeutic target for CRC treatment is particularly intriguing. Given its upregulation in CRC and its role in promoting tumor proliferation and stemness, targeting OLFML1 could disrupt critical pathways driving tumor progression. Therapeutic strategies could include the development of small-molecule inhibitors, monoclonal antibodies, or RNA-based approaches to inhibit OLFML1 expression or function. Inhibition of OLFML1 could synergize with chemotherapy or targeted therapies by disrupting key pathways involved in CSC maintenance, tumor survival, and therapy resistance. OLFML1 supports oncogenic signaling, including the Hedgehog pathway, which drives CSC self-renewal and promotes resistance to conventional chemotherapy and targeted treatments. By blocking OLFML1, CSC populations may be reduced, sensitizing tumors to chemotherapeutic agents like 5-FU or oxaliplatin and preventing recurrence. Additionally, OLFML1 inhibition weakens tumor-stroma interactions in the microenvironment, reducing protective factors that shield cancer cells. In targeted therapies, OLFML1 inhibition could enhance efficacy by shutting down compensatory survival pathways, such as those activated during resistance to EGFR or VEGF inhibitors. This combinatorial strategy may result in improved tumor suppression for aggressive or resistant CRC. Furthermore, as OLFML1 is a secreted glycoprotein, it may be accessible for therapeutic intervention, making it an attractive target for drug development. However, the feasibility and efficacy of such approaches need to be rigorously tested in preclinical and clinical settings. Additionally, the potential off-target effects and toxicity of OLFML1 inhibition must be carefully evaluated to ensure the safety of therapeutic interventions.

In conclusion, this study sheds light on the molecular mechanisms underlying CRC progression and stemness, identifying OLFML1 as a pivotal factor in these processes. By uncovering the regulatory axis involving IGF2BP3, OLFML1, and the Hedgehog signaling pathway, our findings enhance the understanding of CRC biology and provide a foundation for novel therapeutic strategies. The identification of OLFML1 as a potential diagnostic biomarker and therapeutic target highlights the significance of this research in advancing CRC treatment and improving patient outcomes.

## Supplementary Material

Supplementary figures and tables.

## Figures and Tables

**Figure 10:**
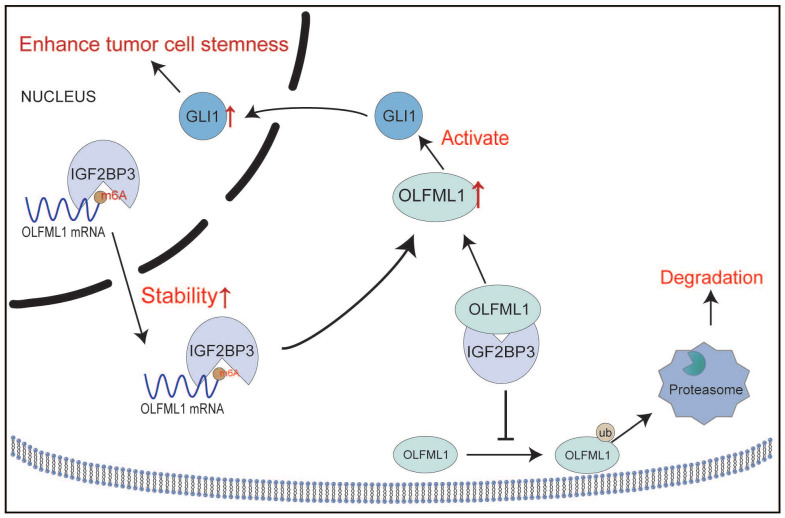
** Schematic diagram demonstrating the mechanism of OLFML1 and IGF2BP3 to enhance CRC stemness.** IGF2BP3 in CRC cells promotes the stability of OLFML1 mRNA by directly binding to it. Additionally, IGF2BP3 inhibits the ubiquitin-proteasome-mediated degradation of OLFML1 by directly interacting with the OLFML1 protein, thereby upregulating OLFML1 expression. OLFML1, in turn, activates GLI1, promoting its nuclear translocation and enhancing the stemness characteristics of CRC cells.

## Abbreviations

CRC: colorectal cancer
CSCs: cancer stem cells
OLFML1: olfactomedin-like 1
IGF2BP3: insulin-like growth factor 2 mRNA-binding protein 3
PMSF: phenylmethylsulphonyl fluoride
RIPA: radioimmunoprecipitation assay
CCK-8: cell counting kit-8
CLIP: crosslinking immunoprecipitation
MeRIP: methylated RNA immunoprecipitation
GEO: Gene Expression Omnibus
IHC: immunohistochemistry
GSEA: gene set enrichment analysis
IP: immunoprecipitation
LC-MS: liquid chromatography-mass spectrometry
Co-IP: co-immunoprecipitation
CHX: cycloheximide
IF: immunofluorescence
m^6^A: N6-Methyladenosine
