# Supplementary material for: Stabilization of OLFML1 via m6A Reader IGF2BP3 Drives CSC Characteristics Through Hedgehog Pathway Activation in CRC
Source: Int J Biol Sci. 2025 Jun 23;21(10):4334–52. doi: 10.7150/ijbs.111032 (PMC12320246; doi:10.7150/ijbs.111032)
Supplement: Supplementary file 1 — Supplementary figures and tables. [file ijbsv21p4334s1.zip › Supplementary Data/Supplementary Table 3.docx]

**Supplementary Table S3（OLFML1+IGF2PB3/74）**

| **Characteristics** | **Non co-high (%)** | **Co-High, n (%)** | | **χ2 value** | ***P value*** |
| --- | --- | --- | --- | --- | --- |
| **Frequency (%)** | 37（50.0） | | 37（50.0） |  |  |
| **Gender, n (%)** |  | |  |  |  |
| Male | 21（50.0） | | 21（50.0） | 0.000 | 1.000 |
| Female | 16（50.0） | | 16（50.0） |  |  |
| **Age, n (%)** |  | |  |  |  |
| ＜55 | 10（58.8） | | 7（41.2） | 0.687 | 0.407 |
| ≥55 | 27（47.4） | | 30（52.6） |  |  |
| **Tumour size (diameter in cm)** | | |  |  |  |
| ＜5 | 24（64.9） | | 13（35.1） | 6.541 | **0.011** |
| ≥5 | 13（35.1） | | 24（64.9） |  |  |
| **Tumour differentiation** | | |  |  |  |
| Good | 6（85.7） | | 1（14.3） | 3.948 | 0.139 |
| Moderate | 26（46.4） | | 30（53.6） |  |  |
| Poor | 5（45.5） | | 6（54.5） |  |  |
| **Depth of tumour invasion** | | |  |  |  |
| Mucosa+muscularis | 18（72.0） | | 7（28.0） | 7.309 | **0.007** |
| Full-thickness | 19（38.8） | | 30（61.2） |  |  |
| **T classification** | | |  |  |  |
| T1 | 8 (100.0) | | 0 (0.0) | 11.234 | **0.011** |
| T2 | 10 (58.8) | | 7 (41.2) |  |  |
| T3 | 12 (36.4) | | 21 (63.6) |  |  |
| T4 | 7 (43.7) | | 9 (56.3) |  |  |
| **N classification** | | |  |  |  |
| N0 | 25 (62.5) | | 15 (37.5) | 5.600 | 0.061 |
| N1 | 9 (37.5) | | 15 (62.5) |  |  |
| N2 | 3 (30.0) | | 7 (70.0) |  |  |
| **M classification** |  | |  |  |  |
| M0 | 36 (50.7) | | 35 (49.3) | 0.347 | 0.556 |
| M1 | 1 (33.3) | | 2 (66.7) |  |  |

**Table S3. Correlation of OLFML1^high^ IGF2BP3^high^ expression with pathological status in 74 cases of patients with CRC.**
