# Supplementary material for: Stabilization of OLFML1 via m6A Reader IGF2BP3 Drives CSC Characteristics Through Hedgehog Pathway Activation in CRC
Source: Int J Biol Sci. 2025 Jun 23;21(10):4334–52. doi: 10.7150/ijbs.111032 (PMC12320246; doi:10.7150/ijbs.111032)
Supplement: Supplementary file 1 — Supplementary figures and tables. [file ijbsv21p4334s1.zip › Supplementary Data/Supplementary Table 2.docx]

**Supplementary Table S2（IGF2BP3/74）**

| **Characteristics** | **Low, n (%)** | **High, n (%)** | **χ2 value** | ***P value*** |
| --- | --- | --- | --- | --- |
| **Frequency (%)** | 30（40.5） | 44（59.5） |  |  |
| **Gender, n (%)** |  |  |  |  |
| Male | 18（42.9） | 24（57.1） | 0.216 | 0.642 |
| Female | 12（37.5） | 20（62.5） |  |  |
| **Age, n (%)** |  |  |  |  |
| ＜55 | 8（47.1） | 9（52.9） | 0.389 | 0.533 |
| ≥55 | 22（38.6） | 35（61.4） |  |  |
| **Tumour size (diameter in cm)** | |  |  |  |
| ＜5 | 19（52.8） | 17（47.2） | 4.355 | **0.037** |
| ≥5 | 11（28.9） | 27（71.1） |  |  |
| **Tumour differentiation** | |  |  |  |
| Good | 5（71.4） | 2（28.6） | 3.422 | 0.181 |
| Moderate | 20（35.7） | 36（64.3） |  |  |
| Poor | 5（45.5） | 6（54,5） |  |  |
| **Depth of tumour invasion** | |  |  |  |
| Mucosa+muscularis | 13（52.0） | 12（48.0） | 2.057 | 0.152 |
| Full-thickness | 17（34.7） | 32（65.3） |  |  |
| **T classification** | |  |  |  |
| T1 | 6 (75.0) | 2 (25.0) | 4.716 | 0.194 |
| T2 | 7 (41.2) | 10 (58.8) |  |  |
| T3 | 11 (33.3) | 22 (66.7) |  |  |
| T4 | 6 (37.5) | 10 (62.5) |  |  |
| **N classification** | |  |  |  |
| N0 | 19 (47.5) | 21 (52.5) | 1.782 | 0.410 |
| N1 | 8 (33.3) | 16 (66.7) |  |  |
| N2 | 3 (30.0) | 7 (70.0) |  |  |
| **M classification** |  |  |  |  |
| M0 | 29 (40.8) | 42 (59.2) | 0.066^a^ | 0.797 |
| M1 | 1 (33.3) | 2 (66.7) |  |  |

**Table S2. Correlation of IGF2BP3 expression with clinico-pathological status in 74 cases of patients with CRC.**

Chi-square test was used to analyze the correlation of IGF2BP3 expression with gender, age, tumour size, tumour differentiation, depth of tumour invasion, TNM classification.
