# Supplementary material for: Stabilization of OLFML1 via m6A Reader IGF2BP3 Drives CSC Characteristics Through Hedgehog Pathway Activation in CRC
Source: Int J Biol Sci. 2025 Jun 23;21(10):4334–52. doi: 10.7150/ijbs.111032 (PMC12320246; doi:10.7150/ijbs.111032)
Supplement: Supplementary file 1 — Supplementary figures and tables. [file ijbsv21p4334s1.zip › Supplementary Data/Supplementary Table 4.docx]

## Supplementary Table S4: List of antibodies and reagents used in the study.

| **Antibody** | **Catalog Number** | **Commercial providers** | **Application** |
| --- | --- | --- | --- |
| OLFML1 | PA5113011 | ThermoFisher | IHC/IP |
| OLFML1 | orb317687 | biorbyt | IF/WB |
| IGF2BP3 | 14642-1-AP | Proteintech | WB/IHC/RIP/IP |
| IGF2BP3 | sc-365640 | SANTACRUZ | IF |
| GAPDH | 60004-1-Ig | Proteintech | WB |
| CD133 | 66666-1-Ig | Proteintech | WB/IF/IHC |
| ABCG2 | 27286-1-AP | Proteintech | WB |
| EPCAM | 66316-1-Ig | Proteintech | WB |
| LGR5 | 30007-1-AP | Proteintech | WB |
| PTCH1 | AF5202 | Affinity Biosciences | WB |
| SMO | sc-166685 | SANTACRUZ | WB |
| His | 10001-0-AP | Proteintech | WB/IP |
| His | 66005-1-Ig | Proteintech | WB |
| FLAG | 20543-1-AP | Proteintech | WB/IP |
| FLAG | 66008-4-lg | Proteintech | WB |
| PE-LGR5 | 373803 | Biolegend | FCM |
| APC-CD133 | 372805 | Biolegend | FCM |
| Ki67 | ZM-0166-1.5 | ORIGENE | IHC |
| GLI1 | UM800063 | ORIGENE | IHC |
