# Supplementary material for: Stabilization of OLFML1 via m6A Reader IGF2BP3 Drives CSC Characteristics Through Hedgehog Pathway Activation in CRC
Source: Int J Biol Sci. 2025 Jun 23;21(10):4334–52. doi: 10.7150/ijbs.111032 (PMC12320246; doi:10.7150/ijbs.111032)
Supplement: Supplementary file 1 — Supplementary figures and tables. [file ijbsv21p4334s1.zip › Supplementary Data/Supplementary Table 1.docx]

**Supplementary Table S1（OLFML1/107）**

| **Characteristics** | **Low, n (%)** | **High, n (%)** | **χ2 value** | ***P value*** |
| --- | --- | --- | --- | --- |
| **Frequency (%)** | 41（38.3） | 66（61.7） |  |  |
| **Gender, n (%)** |  |  |  |  |
| Male | 22（38.6） | 35（61.4） | 0.004 | 0.95 |
| Female | 19（38.0） | 31（62.0） |  |  |
| **Age, n (%)** |  |  |  |  |
| ＜55 | 16（47.1） | 18（52.9） | 1.611 | 0.204 |
| ≥55 | 25（34.2） | 48（65.8） |  |  |
| **Tumour size (diameter in cm)** | |  |  |  |
| ＜5 | 27（50.9） | 26（49.1） | 7.083 | **0.008** |
| ≥5 | 14（25.9） | 40（74.1） |  |  |
| **Tumour differentiation** | |  |  |  |
| Good | 14（70.0） | 6（30.0） | 12.429 | **0.002** |
| Moderate | 24（34.8） | 45（65.2） |  |  |
| Poor | 3（16.7） | 15（83.3） |  |  |
| **Depth of tumour invasion** | |  |  |  |
| Mucosa+muscularis | 28（68.3） | 13（31.7） | 25.269 | **0.000** |
| Full-thickness | 13（19.7） | 53（80.3） |  |  |
| **T classification** | |  |  |  |
| T1 | 15 (88.2) | 2 (11.8) | 30.957 | **0.000** |
| T2 | 13 (54.2) | 11 (45.8) |  |  |
| T3 | 7 (15.9) | 37 (84.1) |  |  |
| T4 | 6 (27.3) | 16 (72.7) |  |  |
| **N classification** | |  |  |  |
| N0 | 30 (54.5) | 25 (45.5) | 12.666 | **0.002** |
| N1 | 8 (22.2) | 28 (77.8) |  |  |
| N2 | 3 (18.8) | 13 (81.3) |  |  |
| **M classification** |  |  |  |  |
| M0 | 38 (37.6) | 63 (62.4) | 0.030 ^a^ | 0.862 |
| M1 | 3 (50.0) | 3 (50.0) |  |  |

**Table S1. Correlation of OLFML1 expression with clinico-pathological status in 107 cases of patients with CRC.**

Chi-square test was used to analyze the correlation of OLFML1 expression with gender, age, tumour size, tumour differentiation, depth of tumour invasion, TNM classification.
