# Supplementary material for: Stabilization of OLFML1 via m6A Reader IGF2BP3 Drives CSC Characteristics Through Hedgehog Pathway Activation in CRC
Source: Int J Biol Sci. 2025 Jun 23;21(10):4334–52. doi: 10.7150/ijbs.111032 (PMC12320246; doi:10.7150/ijbs.111032)
Supplement: Supplementary file 1 — Supplementary figures and tables. [file ijbsv21p4334s1.zip › Supplementary Data/Supplementary Table 5 primer.docx]

### Supplementary Table S5: Primer used in this study.

| Gene | **Forward (5'-3')** | **Reverse (5'-3')** |
| --- | --- | --- |
| OLFML1 | AGCAACGAGGGCATACATTCAAG | CACTTGTGTAGGTCTGACATCTTCC |
| IGF2BP3 | GCCACCATTCGGAACATCACC | GAATAGACTTACAAGCCGCAGAGG |
| CD133 | CTGACAGAGTACAACGCCAAACC | TGAATAGGAAGACGCTGAGTTACATTG |
| GLI1 | AGCCTGAATCTGTGTATGAAACTGAC | CGTGGATGTGCTCGCTGTTG |
| BMI1 | TCCCTCCACCTCTTCTTGTTTGC | CTGGGGCTGTTGCTGGTTCC |
| EPCAM | ACCTCCATGTGCTGGTGTGTG | CCAGTAGGTTCTCACTCGCTCAG |
| OCT4 | GAGAACCGAGTGAGAGGCAACC | CTGGGCGATGTGGCTGATCTG |
| NANOG | GAGATGCCTCACACGGAGACTG | TTGCCTTTGGGACTGGTGGAAG |
| SOX2 | GCCCAGGAGAACCCCAAGATG | GCAGCCGCTTAGCCTCGTC |
| CD44 | CAGCGGCTCCTCCAGTGAAAG | TTCTGTCTGTGCTGTCGGTGATC |
| OLFML1-M2 | GCTCACGTATCGGGTGGAAT | TGCTGTGGCTCTTCTCAAGG |
| siRNA1-IGF2BP3 | GUGAAUGAACUUCAGAAUUTT | AAUUCUGAAGUUCAUUCACCG |
| siRNA2-IGF2BP3 | AGGAAUUGACGCUGUAUAATT | UUAUACAGCGUCAAUUCCUGC |
| OLFML1-homo-288 | GGGAGAUUGACUACAUACATT | UGUAUGUAGUCAAUCUCCCTT |
| OLFML1-homo-867 | GCCAUUUGGUUCUCACAAATT | UUUGUGAGAACCAAAUGGCTT |
| OLFML1-homo-729 | GGACUGUGGAAGAUCGAAUTT | AUUCGAUCUUCCACAGUCCTT |
