# Supplementary material for: Stabilization of OLFML1 via m6A Reader IGF2BP3 Drives CSC Characteristics Through Hedgehog Pathway Activation in CRC
Source: Int J Biol Sci. 2025 Jun 23;21(10):4334–52. doi: 10.7150/ijbs.111032 (PMC12320246; doi:10.7150/ijbs.111032)
Supplement: Supplementary file 1 — Supplementary figures and tables. [file ijbsv21p4334s1.zip › Supplementary Data/supplementary figure legends.pdf]

Supplementary Figure S1. (Related to Fig 1 and Fig 2)

A Analysis of OLFML1 expression in CRC tissues compared with adjacent normal tissues in the CRC microarray profiles GSE21510. **B** Survival analysis of CRC patients with low and high expression of OLFML1 from CRC microarray profiles GSE17537. **C-E** OLFML1 expression in a panel of CRC cell lines at the mRNA level (A) and the protein level (D-E). **F, G** The expression of OLFML1 were detected by western blot (F) and qRT-PCR (G) in OLFML1-overexpressing SW620 cells and RKO cells. **H, I** The expression of OLFML1 were detected by western blot (H) and qRT-PCR (I) in OLFML1-knockdown HCT8 cells and DLD1 cells. **J, K** Dose-response curves show the effects of 5-FU on the sensitivity of vector, OLFML1 overexpression, siControl and siOLFML1 CRC cells (n = 3).

Data information: Graphs report mean ± SD. Significances were assessed using 2-tailed Student's t-test. ****P*< 0.001, ***P*< 0.01, **P*< 0.05.

Supplementary Figure S2. (Related to Fig 4 and Fig 5)

A The GSEA result indicates an enrichment of gene sets related to stemness-related signaling pathway in OLFML1 overexpression group of the CRC microarray profiles GSE20842, GSE35297, GES37178 and GSE37182. **B, C** Real-time q-PCR analysis shows the effects of downregulation of OLFML1on the expression of stem cell markers in CRC cells (n = 3). **D** Western blots experiments show that overexpression of OLFML1 does not activate the notch pathway.

Data information: Graphs report mean ± SD. Significances were assessed using 2-tailed Student's t-test. ****P*< 0.001, ***P*< 0.01, **P*< 0.05.

Supplementary Figure S3. (Related to Fig 6)

A Mass spectrometry analysis revealed the presence of IGF2BP3 in the immunoprecipitation obtained with the OLFML1 antibody. **B** Endogenous OLFML1 and IGF2BP3 were immunoprecipitated in CaCo2 cells.

Supplementary Figure S4. (Related to Fig 7)

A Colony formation assays were performed to determine the effects of IGF2BP3 on the growth of IGF2BP3-overexpressing CRC cells. The number of colonies (> 50 cells) was scored (n = 3). **B** CCK-8 assays were performed to determine the effects of IGF2BP3 upregulation on the proliferation of CRC cells (n = 4).

Supplementary Figure S5. (Related to Fig 8)

A Western blot shows IGF2BP3 protein expression levels in RKO/vector and RKO/OLFML1 cells. **B** Western blot shows OLFML1 protein expression levels in RKO/vector and RKO/IGF2BP3 cells. **C** The degradation of OLFML1 protein in control and OLFML1-overexpressing DLD1 cells following cycloheximide (CHX, 50 μg/mL) treatment was assessed by western blotting. The quantification of the protein level was normalized to that of GAPDH. **D, E** Western blotting was used to examine the expression of OLFML1 and IGF2BP3 proteins in RKO and SW620 cells transfected with siCtrl, siIGF2BP3, or siIGF2BP3 following MG132 treatment (10 μM, 4 h). **F** The prediction of m^6A modification sites on the OLFML1 transcripts RNA sequences by SRAMP (http://www.cuilab.cn/sramp).

Supplementary Figure S6. (Related to Fig 9)

A-D Spearman's correlation analyses were conducted to assess the associations between the expression levels of CD133 and OLFML1 (A, r = 0.450, P = 0.000), CD133 and IGF2BP3 (B, r = 0.349, P = 0.002), GLI1 and OLFML1 (C, r = 0.334, P = 0.040), and GLI1 and IGF2BP3 (D, r = 0.238, P = 0.041) in colorectal cancer (CRC) tissues. Immunohistochemical scores were categorized as follows: 1+ for scores of 1-4, 2+ for scores of 6-8, and 3+ for scores of 9-12.

Data information: Significances were assessed using Spearman's correlation analyses.

Supplementary Figure S7. (Related to Fig 9)

A, B Colony formation assays were performed in CRC cells treated with Vector, IGF2BP3 knockdown, and both OLFML1 overexpression and IGF2BP3 knockdown.

C, D The in vitro limiting dilution assay showed that IGF2BP3 knockdown decreases the formation proportion of CSC spheres, simultaneously overexpression of OLFML1 reverses this effect (n = 12), likelihood ratio test. **E, F** Tumor sphere formation assays indicate that IGF2BP3 knockdown weakens the formation of CSC spheres, simultaneously overexpression of OLFML1 reverses this effect (n = 3).

Data information: Graphs report mean ± SD. Significances were assessed using 2-tailed Student's t-test. ***$P < 0.001$, **$P < 0.01$, *$P < 0.05$.
